# Supplementary material for: Computational Probing the Methylation Sites Related to EGFR Inhibitor-Responsive Genes
Source: Biomolecules. 2021 Jul 16;11(7):1042. doi: 10.3390/biom11071042 (PMC8302001; doi:10.3390/biom11071042)
Supplement: Supplementary file 1 [file biomolecules-11-01042-s001.zip › supplementary Figure.pdf]

Supplementary Material

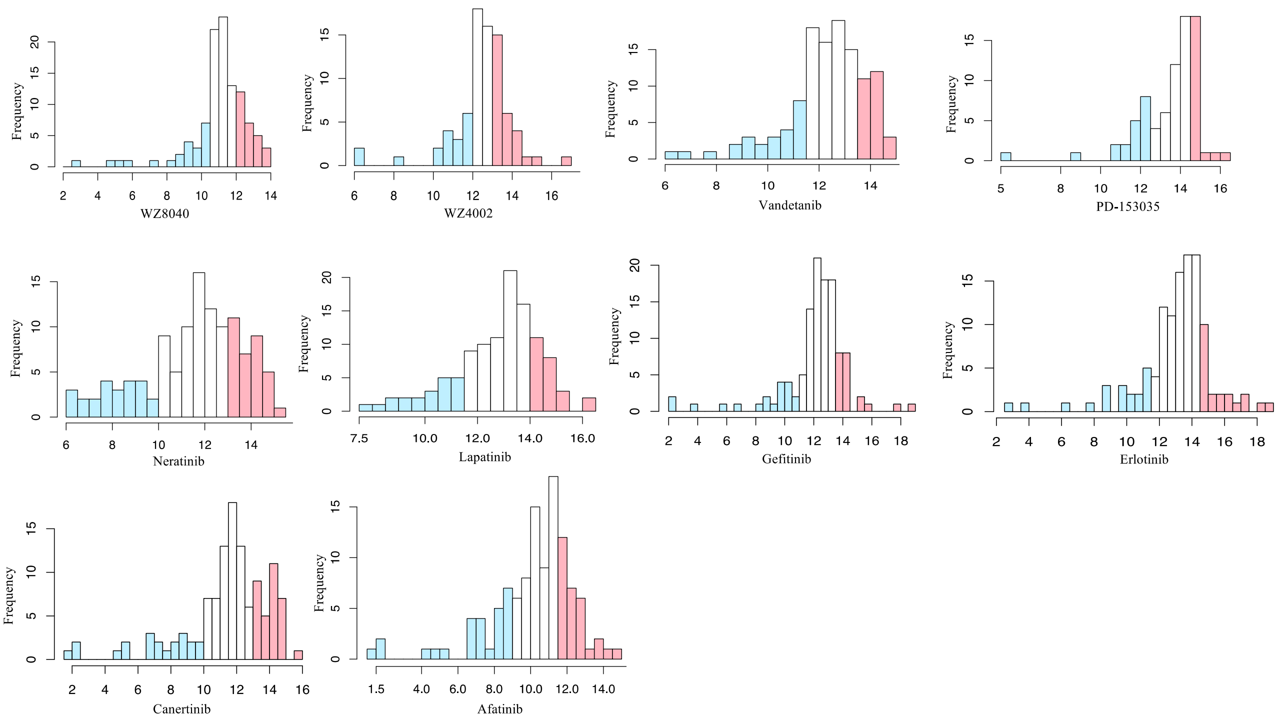

Supplementary Figure S1. According to the experimentally measured AUCDR data of EGFR inhibitors, sensitive or resistant samples were classified, and 10 EGFR inhibitors were considered respectively.
